# Analysis on Artist Neuropsychology and Art Creation

**DOI:** 10.1515/tnsci-2019-0011

**Published:** 2019-04-23

**Authors:** Yaqi Huang

**Affiliations:** 1 Weifang University of Science and Technology, ShouGuang 262700, China

**Keywords:** Neuropsychology, Artistic Creation, extraversion and introverted personality

## Abstract

Based on Carl Gustav Jung’s persona philosophy, this paper conducts theoretical analysis on the introvert and extravert personalities, distinguishes the introvert personality from the psychopathy defined from a medical perspective, and elaborates on the dominance of introvert personality in art creations, as well as the “mental conditions” among artists that the contemporary society is concerned with. This paper uses modern artists as subjects to support the conclusion through analysis of their childhood, career and life stories.

## 1 Introduction

**Figure 1:**
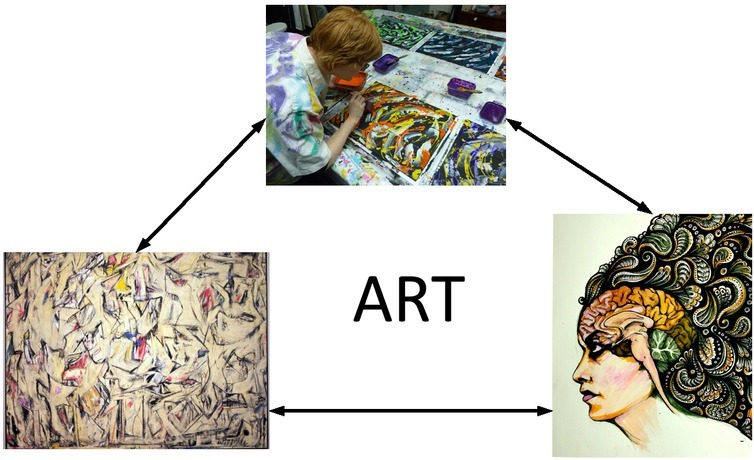
Various manifestations of art

In modern society, and in earlier ages, artists are often called as “psychos”, and their art creation may be called a madman’s action. Artistic works are said to be created when the artist is acting out under mental conditions. In fact, from the physical conditions as human being [1, 2, 3], an artist does not have great differences from the rest of us. But from the perspective of sociology, artists have enhanced social roles to play and social experiences [4, 5, 6]. Their ability in aesthetical appreciation is outstanding, and their expressions of appreciation are dynamic. Figure 1 shows the artistic expressions.

In contemporary society, and even in older times, artists are often referred to as the “sacred disease”. The artist’s creative behaviour will be described as a madman’s behaviour, and the artist’s work will also be said to have been born in an abnormal state of mind. In fact, from the physiological condition of a person, there is not much difference between an artist and an ordinary person. However, from a sociological point of view, an artist has more social roles and experiences than ordinary people. An artist is the subject of an artistic creation activity. He can be an individual or a group. The artist’s production activities mainly tend to be spiritual realms. As the producer of artistic works, the artist’s aesthetic sensibility is extremely developed, and the artistic expression of conveying this aesthetic feeling is also extremely rich.

Why do the artists have advanced sensibility to aesthetics? It means they are particularly sensitive to the things in daily life. They have acute senses and strong interests in the objects they have contact with. The real advantage of artists is their ability to experience and express the meaning and nature via media [7, 8, 9], and to turn these experiences into a physical form that is clear and comprehensible. Art creations originate from the real life. They express the unique personality of the artists, as reflections of external objects on the artist’s psychology ^[10]^. With art works, we sense the unique expression and acute observance of the artists, which is in line with the patterns of artist development ^[11]^.

## 2 Two basic types of personality and functional classification

### 2.1 The distinction between extroverted personality and introverted character

In ancient times, humans repeatedly tried to classify individuals by type. The earliest classification was that the Oriental Astrology Master invented the so-called one-third of the gas, water, earth, and fire in the twelfth house. The “Gang” three palaces, namely the Jade Palace, the Double Uterus, and the Tianping

Palace, form the Qigong group in the Zodiac, while the Lion Palace, the People’s Palace, and the Aries Palace form the Fire Palace. According to this ancient view, people born in the Palace of Fire Palace will show the temperament and destiny of gas or fire. According to this physiological theory and the psychological experiments in Greek medicine, we have four kinds of body fluids, namely biliary type, mucous type, multiple blood type and depression type, so that there are four temperament. We now know that the constellation is the basis for the division of astrology, so what should be used to divide the basic types of psychology? We now gradually answer this question through a psychological experiment. This experiment is like this: there is a small stream, the stream is very wide, but there is no bridge, people can’t walk, they have to jump over, what are they facing? People’s opinions differ greatly from the problems that are too much. We cannot judge whether the decision is correct or not, but what is certain is that everyone has their own methods and habits when making decisions and customer service difficulties. The first person will skip the creek, because he feels it is a fun thing, another person just because he has to go, so he will jump over, the third person, he wants to overcome himself, the customer service is difficult A challenge on the spot, the fourth person, because he couldn’t see what he wanted on the other side, so he didn’t want to jump over. The fifth person didn’t intend to jump over because he felt that jumping over didn’t make sense. Therefore, we have observed that the more natural and negative people are, the more negative they are when they are considered. They will prepare before the action, because it is their habit, so when there is no time to prepare, such negative performance will often lose some opportunities. Another person, he will not consider the action beforehand, but will not make any preparations for rushing to get in. Even if he is already in trouble, he does not know, so they are called “non-thinking type”. “Positive” is more appropriate. However, everyone found that the reason why a person is melancholy is not because he considers more before the action, the other does not consider the first action, and does not necessarily do not know how

to think. The former is because of habitual hesitation, while the latter is self-confident with respect to an object, so you can act without being prepared. Through this observation, we quickly reached the following conclusions. This conclusion is mainly for two types of people. One person has to take a step back when the action has not yet begun. After that, the brain thinking will be on the limbs. In order to be able to respond, such people obviously correspond to the introverted personality, and they have a negative relationship with the object; the other kind of person is the opposite reaction immediately. The personality of a person is corresponding to extroversion, and they are a positive relationship with the object.

**Figure 2:**
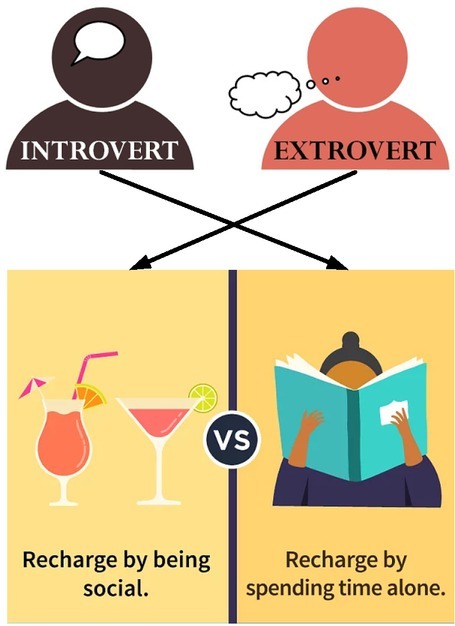
The distinction between extroverted personality and introverted character

A person in depression does not mean that he is depressed from thinking too much before taking actions, or a person does not take actions does not mean he does not think ^[12, 13]^. It maybe that he is hesitant by force of habit in the first case, or that he has confidence in control so no immediate actions need to be taken. For one type of personality, he reverts back before taking actions, and only after that can his brain exert controls over the body to react. This personality is the introverts, who have a negative relation with the objects. The other type of personality can react immediately, those we call the extroverts, who have positive relation with the objects. We can see the differences between introverts and extroverts from Figure 2.

### 2.2 Functional classification of extraversion and introverted personality

After dividing the two basic personalities, the psychologist Jung divides the four basic functions of personality, namely, feeling, intuition, thinking, and emotion. These four psychological functions are the genes of personality, with introversion and extroversion. The characters together constitute four types of introversion and four extroversions.

Functional classification of the extroverted personality:

1) Extraversion sense: People who belong to this type are mostly people who have great interest in accessible reality and who do not have a tendency to reflect and dominate. The life they need is the accumulation of experience in the objective things that touch their senses. The more prominent the character of such people, the higher the demand for sensory. The purpose of their regular treatment of people or things is to have feelings, perceptual objects, and sometimes even more to enjoy the feelings. The inner things are annoying and extremely disgusting in the eyes of such people.
2) Extraversion Intuition: This type of person has a keen sense of the outside world, and they have extremely sensitive discovery and insight into people or things with potential ability and development. Such people can find all kinds of new possibilities in the world. The people who belong to this extraversion instinct are generally people who are innovative, but the behaviour of such people is generally based on intuition, so they are right The understanding of human beings cannot be objectively understood and analysed, which leads to the inability of this type of person to have a persevering psychology.
3) Extroverted thinking: This type of person, they have their own standards and basis for treating people or things, and the ideological characteristics are based on objective facts. They need to stimulate their thinking through outside information. This type of person lacks personality; emotion is not exposed, and sometimes even shows arrogant and cold personality. Scientists are generally extroverted thinking.
4) extroversion emotional type: This type of person, focusing on the expression of emotional function, they are the most obvious in the choice of love, the internal factors are not the scope of their consideration, and the external factors such as identity, age, etc. are their mate choice conditions. . Extroverted emotional people are fond of communication, emotional exposure, and their emotions are in line with universal values ​and objective conditions.

Functional classification of introverted personality:

1) Inwardly inclined type: This type of person pays attention to the feeling of subjective world. They often stay away from the external objective world. Compared with those who are extroverted, their sources of perception are completely opposite. People who feel inwardly are deeply influenced by psychological conditions, and everything seems to come from the depths of their hearts.
2) Introverted intuition: people with introverted intuition, good illusions, no interest in outsiders or things, they often do not start from reality, the various possibilities of the outside are from the thoughts of such people. Therefore, their thoughts and dispositions are often very strange. Jung believes that most artists belong to this type.
3) Introverted thinking: This type of person, their mode of thinking is biased towards rationality, while thinking about the outside world is also carrying out inner self-examination. The emotions of people of this type are oppressive, often obsessed with self, stubbornness, reluctance, and treating people or things with their indifference and showing pride in character. Jung believes that philosophers fall into this category.
4) Introverted Emotional Type: People of this type, their emotions are often hidden in the heart, not exposed, habitually hiding themselves, trying to establish a protective barrier for themselves. They are silent, emotions are repressed, and only their own subjective factors can stimulate their emotions. Therefore, the temperament of such people is often melancholy.

**Figure 3:**
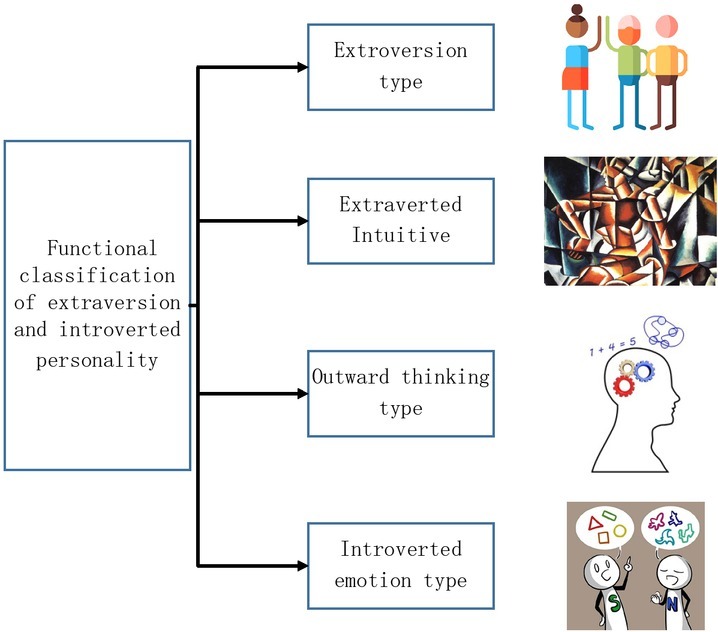
Functional classification of extraversion and introverted personality

Jung further classifies four basic functions, on the basis of two personalities. The functions are sense, intuition, thinking and feeling, which constitute the basis of personality ^[14]^. Coupled with introverted personality and extraverted personality, there are eight personality types including four extroversion types and four introversion types ^[15]^. The extraverted sensing type mostly has great interests in the tangible reality, but no reflection tendency or domination. The extraverted intuitive type has acute sensitivity to the externality, and great intuition and observation of people or objects with potentials ^[16]^. The extraverted thinking types has their own sets of standards for treating people and objects and tends to benchmark mind-sets to the objective facts. They need external information to simulate thinking. The introverted emotion type would like to keep it to them, and are reluctant to expose them, so they build a protection barrier for themselves. The classification is listed in Figure 3.

### 2.3 Abnormal psychology in the emotional personality of an artist

Artist is a social attribute. They do not have discrepancy from the regular people in terms of social attribute, given that artists are also individuals or groups with flesh and blood. But when it comes to the emotional expression, the general public and artists have significant differences. The regular people can find rational ways to convey and express the feelings, while the artists, before emerge of the emotions ^[17, 18]^, are isolated and viewed by the public as aliens. When they have the actual needs for emotional expressions, they may overdo it so that the artists are not easily accepted by the world. And sometimes they are even viewed as abnormal. But our time calls for the uniqueness, and artists too for art creation. The types of works are included in Table 1.

**Table 1:** Different type of works of the artist

Index	2016	2017
Literature	0.071	0.072
Painting	0.757	0.786
Music	0.928	0.951
Dance	0.913	0.945
Sculpture	0.872	0.792
Drama	0.317	0.325
Building	0.909	0.856
The film	0.603	0.751
Abstract art	0.093	0.122
Modern dance	0.509	0.285

## 3 Causes of artists’ introverted psychology and their representative artists

### 3.1 Childhood experience is the cornerstone and decisive factor of introverted psychology

Childhood experience refers to the collective of the experiences when one is in childhood and the psychological experiences from the childhood experiences. All the experience, emotion, knowledge, and memory from the childhood are parts of the childhood experience. It marks the beginning of a psychological journey moving towards maturity, and determines the direction of one’s personality, mind-set, and character as an adult. Also since that man establishes perceptions and accumulation on things when he is young, childhood is an important stage to one’s development.

**Figure 4:**
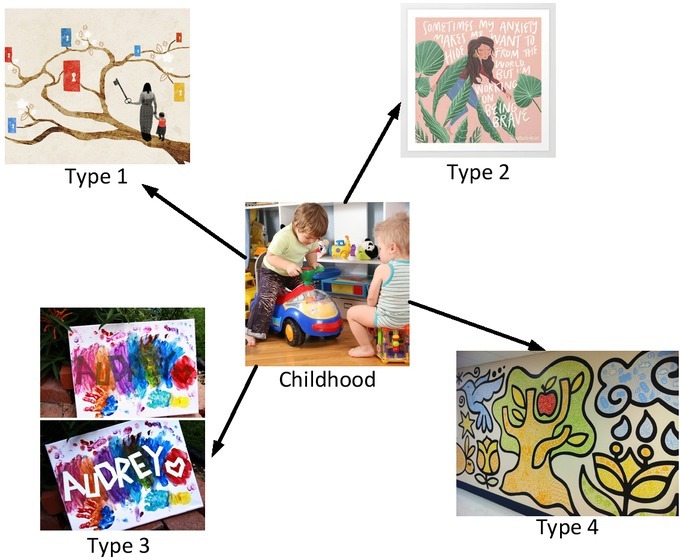
The artistic expression of many forms in childhood

Lack of childhood experiences, for the artists, may be the drive of creation and source of inspiration. They are nourished from it to fuel artistic creativity over a lifetime. A piece of good art is related to the real life. When artists reflect on their defected unfortunate childhood, they feel the pains but also sense the pleasure in putting the life stories into art. Figure 4 shows expressions of childhood.

### 3.2 Suffering events in growth experience are part of introverted psychology

Van Gogh, as an example, longs for love, for a simple life with companionship of family. But he

ended up dying in poverty. In his portraits, he is painted, emaciated and depressed, as a loser in life. For him, selflessness from his younger brother is something he could not repay even in a lifetime. Though people wish they can be an exception, the agony and loneliness is beyond our imagination for those unique artists. It is a sad life journey for Van Gogh, solitude yet great, which made him through the ups and downs of reality and dreams.

Yayoi Kusama, without doubt, is a person with mental conditions. She capitalizes on it for her art creation, and believes that her conditions have made her. From a random piece of her work, installation or space are decorated with great many dots, such as the dotted pumpkin and vase. These paintings have enabled us to understand decorative structures. Different from other artists, her work has oriental mysteriousness and a touch of neurotics. Though they may seem simple, it is the neurotics and crooked actions of the artist that drive her to be one of the top art legends. Her work therefore carries extraordinary meanings.

### 3.3 Data analysis

We analysed the neuropsychological conditions and their art creations with results shown in Table 3.

**Table 2:** The influence of growth experience on artists

Art achievement	Artistic output	Average artwork production time (day)	Future development prospects (year)
15	30	14.1	12
17	50	12.3	11
20	50	11.8	7
21	50	10.5	7
23	60	9.5	5
23	60	8.1	4
24	70	7.8	5

**Table 3:** Data analysis of artists’ neuropsychology and artistic creation in history

Parameter	Artists’ neuropsychology		Artistic creation	
	Type 1	Type 2	Type 1	Type 2
Correlation coefficient	0.9925	0.9802	0.9765	0.9614
Proportion	0.5121	0.5012	0.5221	0.4951

From this analysis, we know that the neuropsychological conditions have high relevance to art creation. The relevance further drives the psychological progress and enhances art creation capacity in artists. For the purpose of analysis, we then classify the neuropsychological conditions and art creation in Table 4.

**Table 4:** Artists categorize according to neuropsychology and artistic creation.

Parameter	Artists’ neuropsychology			Artistic creation		
	Artist 2	Artist 3	Artist 4	Artist 2	Artist 3	Artist 4
Type 1	415	298	300	485	424	417
Type 2	435	400	395	386	291	298
Type 3	-	172	174	-	154	158
Type 4	-	-	0	-	-	0

From Table 2, it is clear the first type has the higher number of artists, who share similar neuropsychological conditions and art creation capacity. The fourth type has lower number of artists. Those in this category, with outstanding neuropsychological conditions and art creation capacity, are among the rare cases. We then perform predictions on the artist behaviour using the analysis method. See results in Table 5.

**Table 5:** Analysis of artist’s mental psychology and artistic creation prediction

Time	Predictive value	95% confidence interval	
2017/6	0.944 9	0.326 1	1.563 8
2017/7	0.955 7	0.336 8	1.574 5
2017/8	1.000 9	0.381 7	1.620 1
2017/9	0.987 9	0.368 4	1.607 3
2017/10	0.973 9	0.341 5	1.606 3

**Figure 5:**
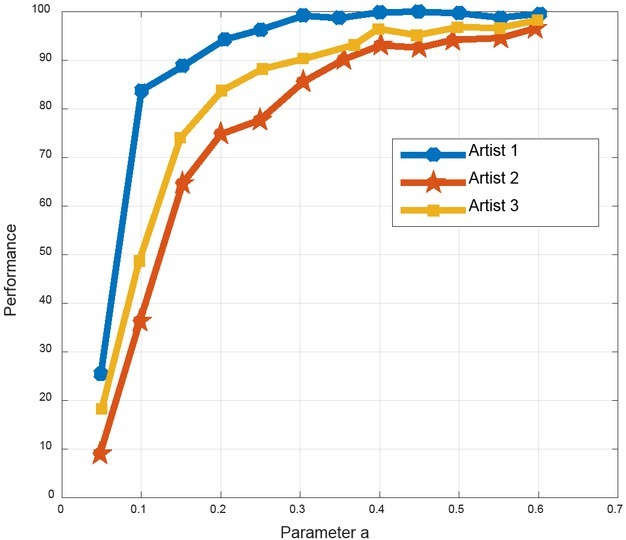
Analysis of the performance of different artists

From Table 5, it is clear our analysis method has good predictability of artist behaviour with satisfactory results. It has practical utilization. In the meantime, the performance results also vary among artists with results shown in Figure 5. We know from this fig that artist 1 has the best performance.

The emerging force of the new drives the advances of time and human race. It will take a long time before we fully understand the neuropsychology of artists, but I believe that

as long as art exists, and those live for art live, neuropsychology of artists will someday be recognized by the general public. With this recognition, art will gain new momentum for further development.

## 4 Conclusions

The neuropsychological conditions demonstrated by artists in art creations should be subject to rational analysis and proper assessment. The so-called “abnormal state” is the driving force of art creation, drastically different from the “abnormality” by medical meaning. From the underlying content and form, it is also inconsistent with the traditional sense of abnormal. Being an ultimate form of appreciation, art sets to express the true, and the beautiful rather than the ugly or the meaningless. The states of artists are the precondition of sensing, expressing, and realizing themselves and a necessary emotional demand for art creation, and an ultimate mind-set and psychological state for them to realize arts. The emerging force of the new drives the advances of time and human race. It will take a long time before we fully understand the neuropsychology of artists, but I believe that as long as art exists, and those live for art live, neuropsychology of artists will someday be recognized by the general public. With this recognition, art will gain new momentum for further development.
